# In *S. cerevisiae* hydroxycitric acid antagonizes chronological aging and apoptosis regardless of citrate lyase

**DOI:** 10.1007/s10495-020-01625-1

**Published:** 2020-07-14

**Authors:** Maurizio D. Baroni, Sonia Colombo, Olivier Libens, Rani Pallavi, Marco Giorgio, Enzo Martegani

**Affiliations:** 1 Department of Biology, University of Padua, Padua, Italy; 2 Department of Biotechnology and Biosciences, University of Milano-Bicocca, Milan, Italy; 3 Department of Experimental Oncology, European Institute of Oncology (IEO), Milan, Italy; 4 Department of Biomedical Sciences, University of Padua, Padua, Italy

**Keywords:** Caloric restriction mimetics, Hydroxycitric acid, Aging, Apoptosis/necrosis, Oxidative stress, Sch9 and Ras2 pathways

## Abstract

Caloric restriction mimetics (CRMs) are promising molecules to prevent age-related diseases as they activate pathways driven by a true caloric restriction. Hydroxycitric acid (HCA) is considered a bona fide CRM since it depletes acetyl-CoA pools by acting as a competitive inhibitor of ATP citrate lyase (ACLY), ultimately repressing protein acetylation and promoting autophagy. Importantly, it can reduce inflammation and tumour development. In order to identify phenotypically relevant new HCA targets we have investigated HCA effects in *Saccharomyces cerevisiae*, where ACLY is lacking. Strikingly, the drug revealed a powerful anti-aging effect, another property proposed to mark bona fide CRMs. Chronological life span (CLS) extension but also resistance to acetic acid of HCA treated cells were associated to repression of cell apoptosis and necrosis. HCA also largely prevented cell deaths caused by a severe oxidative stress. The molecule could act widely by negatively modulating cell metabolism, similarly to citrate. Indeed, it inhibited both growth reactivation and the oxygen consumption rate of yeast cells in stationary phase. Genetic analyses on yeast CLS mutants indicated that part of the HCA effects can be sensed by Sch9 and Ras2, two conserved key regulators of nutritional and stress signal pathways of primary importance. Our data together with published biochemical analyses indicate that HCA may act with multiple mechanisms together with ACLY repression and allowed us to propose an integrated mechanistic model as a basis for future investigations.

**Electronic supplementary material:**

The online version of this article (10.1007/s10495-020-01625-1) contains supplementary material, which is available to authorized users.

## Introduction

The reduction of calorie intake without malnutrition can extend health span in both model organisms and humans by lowering the incidence of age-related diseases. Caloric restriction mimetics (CRMs) can activate some protective pathways driven by a true caloric restriction leading to a beneficial modulation of several cell processes (reviewed in [1]).

Some bona fide CRMs are nutraceuticals, such as salicylate and hydroxycitric acid, HCA [1]. Aspirin/salicylate can increase lifespan of model organisms and are considered cancer prevention molecules [2–4]. They inhibits EP300 and protein acetylations and can activate autophagy [5–7]. In MnSOD-deficient yeast cells aspirin/salicylate starve the mitochondria of acetyl-CoA, inducing cell death [8]. In *WT* yeast cells salicylate can alter the balance between cell quiescence, death and proliferation as a function of metabolic signals [9]. Hydroxycitric acid, usually extracted from *Garcinia cambogia* [10], is widely used as a diet supplement for weight reduction [11]. *Garcinia cambogia* extracts and/or HCA appear to have a low or negligible impact in terms of acute or chronic toxicity, genotoxicity, reproductive failure and teratogenicity. Although sporadic cases of mild or more severe adverse reactions have been registered [12], specific studies in humans have shown no significant differences about several side effects or adverse events between treated individuals and controls at the recommended doses [13–15]. Rather, HCA shows several beneficial pleiotropic effects in mammals. It decreases lipogenesis, insulin resistance, inflammation and oxidative stress; it also promotes autophagy and the efficacy of some antitumor therapies (reviewed in [1]). HCA is thought to act mainly as competitive inhibitor of ATP-dependent citrate lyase (ACLY), cleaving citrate to oxaloacetate and AcCoA [16–19]. Indeed, the AcCoA produced by ACLY is crucial for the metabolism of fatty acids, the biosynthesis of cholesterol, the acetylation and prenylation of proteins and gluconeogenesis (reviewed in [20]). Of note, HCA also appears to increase the concentrations of endogenous serotonin and upregulate serotonin receptor genes, these mechanisms likely contributing to satiety, fat oxidation and decreased de novo lipogenesis [15, 21, 22].

Importantly, ACLY expression and activity has been found to be aberrantly expressed in many tumours (e.g. glioblastoma, colorectal cancer, breast cancer and others) and its inhibition correlated to repression of tumour proliferation and apoptosis. So, ACLY is considered an anti-cancer drug target with a great therapeutic potential [20, 23, 24], and there is a great effort to develop new ACLY inhibitors or to re-evaluate those previously developed for metabolic disorders [20, 25–27]. In this therapeutic context, the knowledge of new targets modulated by ACLY inhibitors, such as HCA, could be of pivotal importance.

The budding yeast *Saccharomyces cerevisiae* gives the unique opportunity to identify new, physiologically relevant, in vivo targets of HCA since this model organism naturally lacks ACLY [28, 29]. Indeed, our phenotypic analyses show that HCA can strongly antagonize chronological aging, apoptosis/necrosis and ROS-induced cell death in the absence of ACLY, likely acting with multiple mechanisms involving metabolism as well as nutritional and stress signal pathways. Our findings together with published data has also allowed to conceive an integrated multi-target HCA mechanistic model to be tested in future biochemical and molecular studies.

## Results

### HCA inhibited chronological aging and age-related apoptotic/necrotic processes

**Fig. 1:**
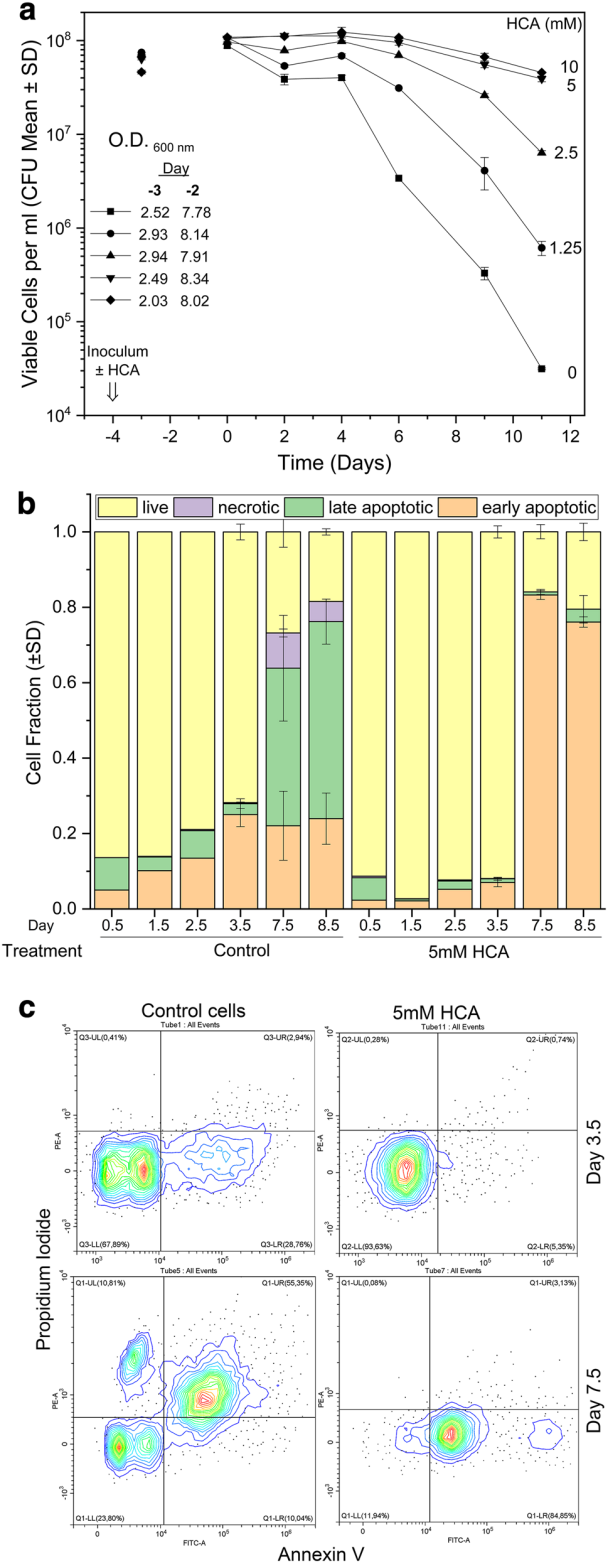
In budding yeast hydroxycitric acid strongly antagonized cell chronological aging, apoptosis and necrosis despite the absence of ACLY. **a** Cell survival of *S. cerevisiae* cells was monitored with colony forming units during 11 day CLS, in the presence of different concentrations of HCA (given at the moment of inoculation). Population cell densities were also followed at days − 3 and − 2 as OD_600_ values, as reported. **b **and** c** Quantitative analysis of live, early apoptotic, late apoptotic and necrotic yeast cells after Annexin V/propidium iodide staining of chronologically aging yeast populations treated or not with 5 mM HCA. Samples (n = 3) were taken from day 0.5 to 8.5 CLS (**b**). Two representative examples of flow cytometric analyses performed on days 3.5 and 7.5 CLS ± 5 mM HCA. Quadrants Q1–4 were constructed based on unstained samples (not shown). Lower left (LL), lower right (LR), upper right (UR) and upper left (UL) quadrants Q contain live (Ann V^−^/PI^−^), early-apoptotic (Ann V^+^/PI^−^), late apoptotic (Ann V^+^/PI^+^) and necrotic (Ann V^−^/PI^+^) cells, respectively (**c**)

The yeast *S. cerevisiae* is a model for eukaryotic cell and can provide significant insights into the human biology of aging [30–32]. It has been suggested that bona fide CRMs should also have the capacity to extend lifespan in model organisms [1]. Indeed, some CRMs, such as spermidine or rapamycin, can prolong CLS in yeast [33, 34]. So we decided to monitor yeast aging in the presence of HCA. Cells of W303-1A (*WT*) strain were inoculated (day − 4 of experiments) in synthetic glucose medium with variable concentrations of hydroxycitric acid (0–10 mM). After growth there was no further increase in cell numbers and OD values between day − 2 and 0 (Fig. 1a inset and data not shown). Cell number at day 0 was about 10^8^ cells/ml in all conditions. Then the progressive loss of viability of these stationary phase cells kept in their culture medium was monitored from day 0 until day 11 and used as an index of chronological aging [35] (Fig. 1a). Strikingly, even in the absence of its known target ACLY, HCA showed a potent anti-aging activity. Indeed, it strongly prolonged CLS, acting in a concentration dependent manner until an apparent saturation effect at dosages higher than 5 mM. At these dosages after 6 days all HCA treated population was still alive whereas control cell viability dropped to 4%; at day 11 CLS there was about 50% or 0.04% of surviving cells with or without HCA, respectively (Fig. S1). As a control condition, stationary phase cells were shifted in water at day 0 where, as expected [35], all populations retained full viability throughout the experiment (Fig. S2).

Yeast chronological aging is coupled to the accumulation of apoptotic/necrotic cell subpopulations and other phenotypes [36] intriguingly similar to the hallmarks of human aging [32]. In order to monitor yeast apoptosis/necrosis during CLS under the effects of hydroxycitric acid, two parallel W303-1A cell cultures were incubated with or without 5 mM HCA (added at the moment of the inoculum) and left to age for almost 9 days; meanwhile, the cells were double stained with Annexin V and propidium iodide and analysed by flow cytometry [36] (Fig. 1b, c). Over the first 4 days there was a gradual increase of apoptotic cells in untreated populations that was almost absent in HCA treated cells. A dramatic accumulation of late apoptotic/necrotic cells was then seen after 7.5 or 8.5 days in control culture but not in the HCA treated cells. Rather, these latter ones showed a great increase in Annexin V^+^/PI^−^cells (see also Fig. S3), considered to be in early apoptosis. This apparent 70–80% of early apoptotic cells was surprising considering that ≥ 50% of HCA-treated cells were still able to form a colony (Fig. 1a). This partial discrepancy likely revealed that in some cells the early apoptotic processes, monitored here only via phosphatidyl serine exposure, were still reversible when they are plated on YEPD. Anyhow HCA was clearly able to fully prevent the age-associated massive increase of late apoptotic/necrotic cell deaths (Fig. 1b, c).

### HCA rescued yeast from cell death induced with different pro-apoptotic agents

**Fig. 2:**
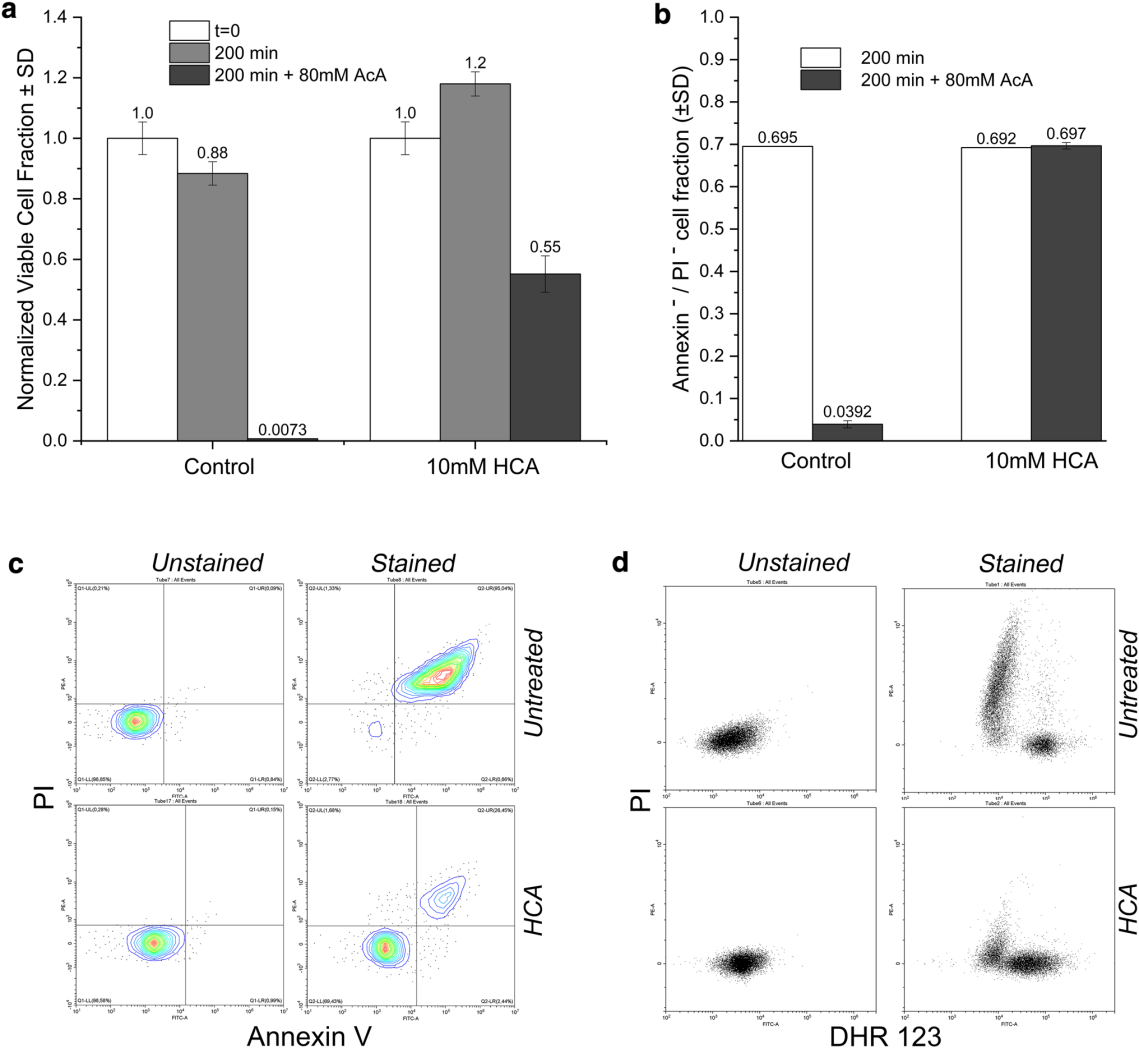
Hydroxycitric acid rescued yeast cells from both acetic acid induced apoptosis and cell death caused by a severe oxidative stress. **a–c** Cell viability determined after a pro-apoptotic treatment with acetic acid (AcA) of exponentially growing cells, in the presence or not of 10 mM HCA. CFUs were monitored after incubation of cell at pH 3 ±10 mM HCA for 200 min with or without 80 mM AcA. CFU have been normalized on untreated cells at time 0 (absolute CFU are reported in Fig. S4). n = 3 for each condition (**a**). Live cells were also determined as Annexin V^−^/PI^−^ fraction ± 10 mM HCA in the presence or not of 80 mM acetic acid (conditions as before). n = 3 (**b**). Representative example of flow cytometric analysis of Annexin V/PI stained cells showing HCA rescue effect from apoptosis induction with acetic acid. Q_LL_= live cells, Q_LR _= early-apoptotic cells, Q_UR _= late-apoptotic cells and Q_UL _= necrotic cell (**c**). **d** A representative flow cytometric analysis of dihydrorhodamine 123/propidium iodide double stained *WT* yeast cells collected after exposure to 70 mM H_2_O_2_ for 20 min. Cells were grown from the inoculum in the presence or not of 10 mM HCA and collected after 3 day CLS. Different cell classes were identified and quantified by manual gating as described in Fig. S5

Acetic acid (AcA) accumulated in the culture medium has been proposed to partially contribute to the mechanisms of yeast chronological aging [37]. On the other side, a treatment with AcA can induce different pathways of programmed cell death independently of chronological aging [38]. So, in order to investigate a possible more general anti-apoptotic role of HCA, we analysed the behaviour of exponentially growing cells subjected to a pro-apoptotic treatment with acetic acid [39]. W303-1A cells exponentially growing in SD ± 10 mM HCA were harvested and incubated in the same medium at pH 3.0 (see “Materials and methods” section) containing or not 80 mM acetic acid. Thereafter the cell viability (as CFUs) and the apoptotic behaviour of cells (seen by Annexin V/PI staining) was monitored until 200 min (Fig. 2a–c; Fig. S4), a time interval sufficient to irreversibly drop cell viability to 1–3% (while no significant effect was caused by low pH per se, as described [39]). In particular, AcA treatment was associated to the accumulation of late apoptotic cells in almost the entire population. In sharp contrast, most HCA-treated cells (60–70%) were still able to form a colony and resulted to be Annexin V^−^/PI^−^ (whereas only 25% of the population underwent late apoptosis). These findings clearly show that there was an efficient HCA rescue activity from AcA-induced apoptosis independently of chronological aging.

A severe oxidative stress can lead to a massive ROS production possibly followed by cell death [40]. *S. cerevisiae* plays a key role in understanding of the relationships between aging and the non-homeostatic ROS production, given that the mammalian and yeast antioxidant responses are similar [41]. So, it appeared relevant to study the response of HCA-treated aging yeast cells to H_2_O_2_ induced oxidative stress. To this aim both ROS and accumulation of dead cells were followed by staining chronologically aged (day 3 CLS) *WT* yeast cells with DHR 123 and PI, after a H_2_O_2_ treatment in the presence or not of 10 mM HCA. Strikingly, the molecule was able to largely prevent the massive cell death (PI^+^ cells) caused by the intense oxidative stress. In parallel there was a sharp increase of live cells with high ROS levels (PI^−^/DHR123^+^ cells) and a much less pronounced increase of DHR123^−^ live cells (Fig. 2d; Fig. S5 for a quantitative analysis). Results after 1 or 2 days of aging were also consistent with these conclusions (data not shown).

### HCA interacted with genetic elements affecting CLS

**Fig. 3:**
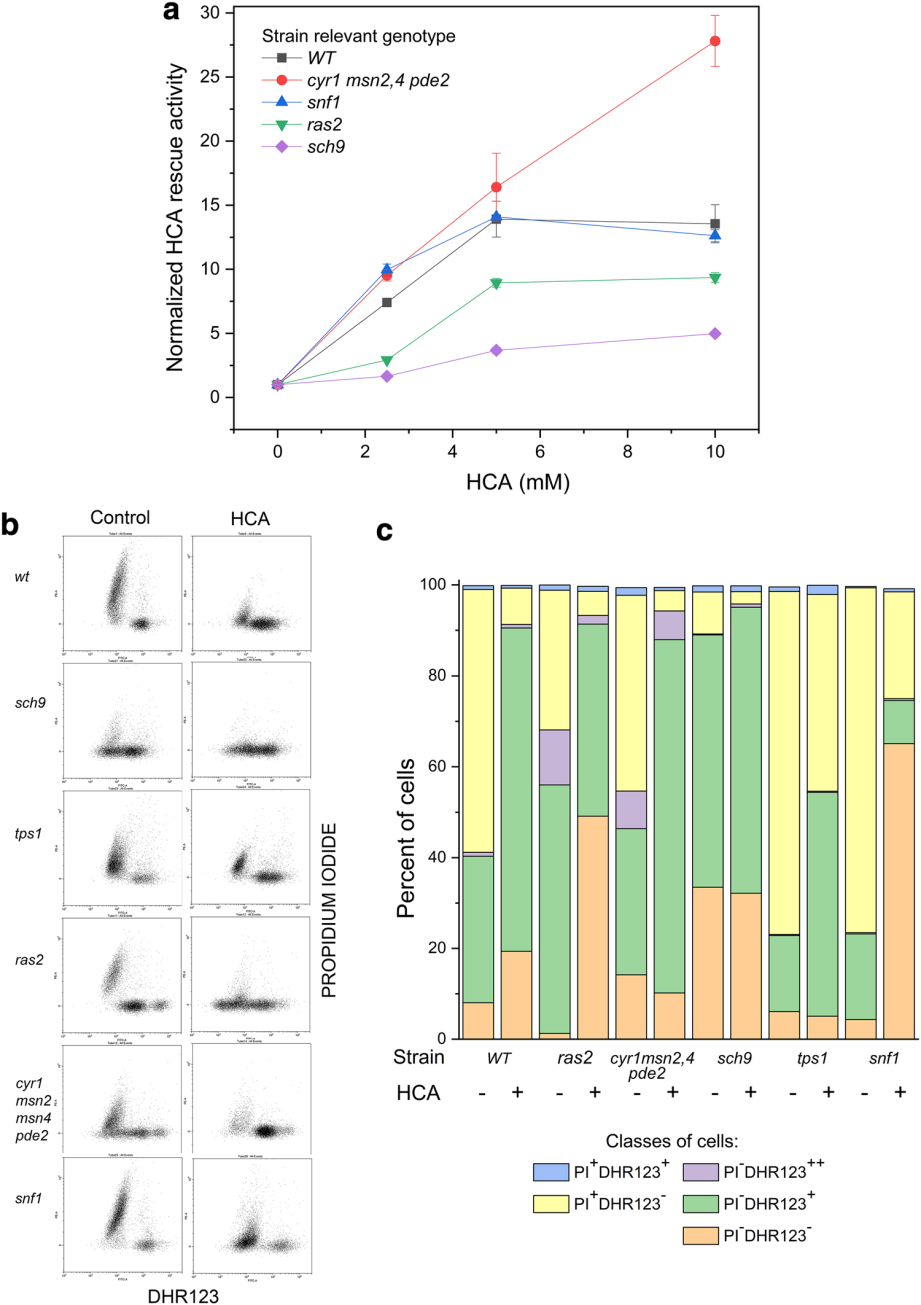
CLS mutants responded differently to HCA during aging or oxidative stress. **a** Comparison of HCA rescue activity among CLS mutants as a function of HCA dosage, during chronological aging. CFU were collected after 6 days of aging, then the cell survival fraction of *WT* and each mutant strain, treated with different HCA doses, was calculated respect to the untreated control, taken as 1 (n = 2). **b **and** c** Representative cytofluorimetric analyses of DHR123/PI stained mutant cells after a severe H_2_O_2_ driven oxidative stress in the presence or not of 10 mM HCA (same conditions described in Fig. 2d) (**b**). Different cell classes were identified by manual gating and quantified: live cells not producing ROS (PI^−^DHR123^−^), live cells producing high ROS levels (PI^−^DHR123^+^), live cells producing very high levels of ROS (PI^−^DHR123^++^), dead cells (PI^+^DHR123^−^) and a very minor fraction of population consisting of dead or dying cells apparently with high ROS levels (PI^+^DHR123^+^) (n = 2) (**c**)

To test the idea that HCA could impinge on signal pathways controlling aging, we performed some analyses in cells with deletions of genes affecting CLS (listed in Table 1). We confirmed the aging phenotypes of the mutants (data not shown), then the viability (as CFUs) of each of them were monitored after 6 days of chronological aging in the presence of different HCA concentrations (Fig. S6). Based on these data, the increase of the cell survival fraction was calculated and normalized to 1 for the untreated population of each mutant, so that the effect of the nutraceutical could be directly compared among strains (Fig. 3a). As expected the rescue activity of HCA increased in a dose dependent way until a concentration of 5 mM, with one exception (discussed in Fig. S7 legend). The results (Fig. 3a; Fig. S7) clearly show that *ras2* and, more pronouncedly, *sch9* cells resulted to be less responsive to the HCA rescue activity. These results were confirmed by following complete aging kinetics of mutants with or without 10 mM HCA (Fig. S8). In contrast, both *snf1* and *cyr1msn2msn4pde2* mutants responded similarly to WT cells, if not even better. Since in the quadruple mutant [42] both cyclic-AMP and Msn2 and 4, two master regulators of stress-responsive genes inhibited by cAMP-dependent PKA, are dispensable for HCA rescue effects, a possible Ras2 contribution to HCA phenotype could not be mediated by cAMP-PKAs.

**Table 1:** List of yeast strains used in this study

Relevant genotype	Source/reference	Abbreviation used in text and figures	Original name
*MATa ade2-1 can1-100 his3-11,15 leu2-3112 trp1-1 ura3-1*	[70]	*WT*	W303-1A
*ras2::URA3*	Our laboratory	*ras2*	SC592
*sch9::NATMX2*	J. Winderickx (KU Leuven)	*sch9*	JW03083
*snf1:HIS3*		*snf1*	JW03963
*cyr1::KanMX2 msn2::HIS3 msn4::TRP1 pde2::TRP1*	[42]	*cyr1 msn2,4 pde2*	SC104
*tps1:TRP1*	[71]	*tps1*	YSH290

All strains are isogenic to W303-1A

After analysing the same mutants (together with a *tps1* mutant) subjected to a treatment with H_2_O_2_, we could establish that some of them responded differently to HCA also during a severe oxidative stress. Figure 3b shows cytofluorimetric analyses of DHR123/PI stained mutant cells aged for 3 days in the presence or not of 10 mM HCA and then treated with hydrogen peroxide. As described above (Fig. S5) five classes of cells were distinguishable and were quantified after discharging most cell doublets (coincidences) (Fig. S9). In all mutants the rescue effect of HCA was recognizable, although at different degrees, and three different types of response were observable (Fig. 3b, c; Fig. S10): (a) in the case of *cyr1msn2msn4pde2* and *tps1* mutants the response to HCA was similar to that of *WT*, namely there was mainly a strong decrease of PI^+^ cells and a corresponding increase in viable cells producing high levels of ROS; of note, in *tps1* cells the HCA rescue activity appeared less efficient; (b) In the case of *ras2* and *snf1* cells there was a similar large increase in viable cells driven by HCA but these rescued cells did not produce high ROS levels. Interestingly, it appeared that not only PI^+^ cells but also live cells producing high or very high ROS levels cells decreased as a result of the HCA treatment; (c) finally, *sch9* mutant responded very partially to the molecule. Indeed, these cells were already very resistant to H_2_O_2_ treatment, so mimicking the HCA effect. However, a small additional increase of live (ROS producing) cells induced with HCA was reproducibly measurable.

### HCA could control yeast growth as a function of cell physiology and inhibited respiration

**Fig. 4:**
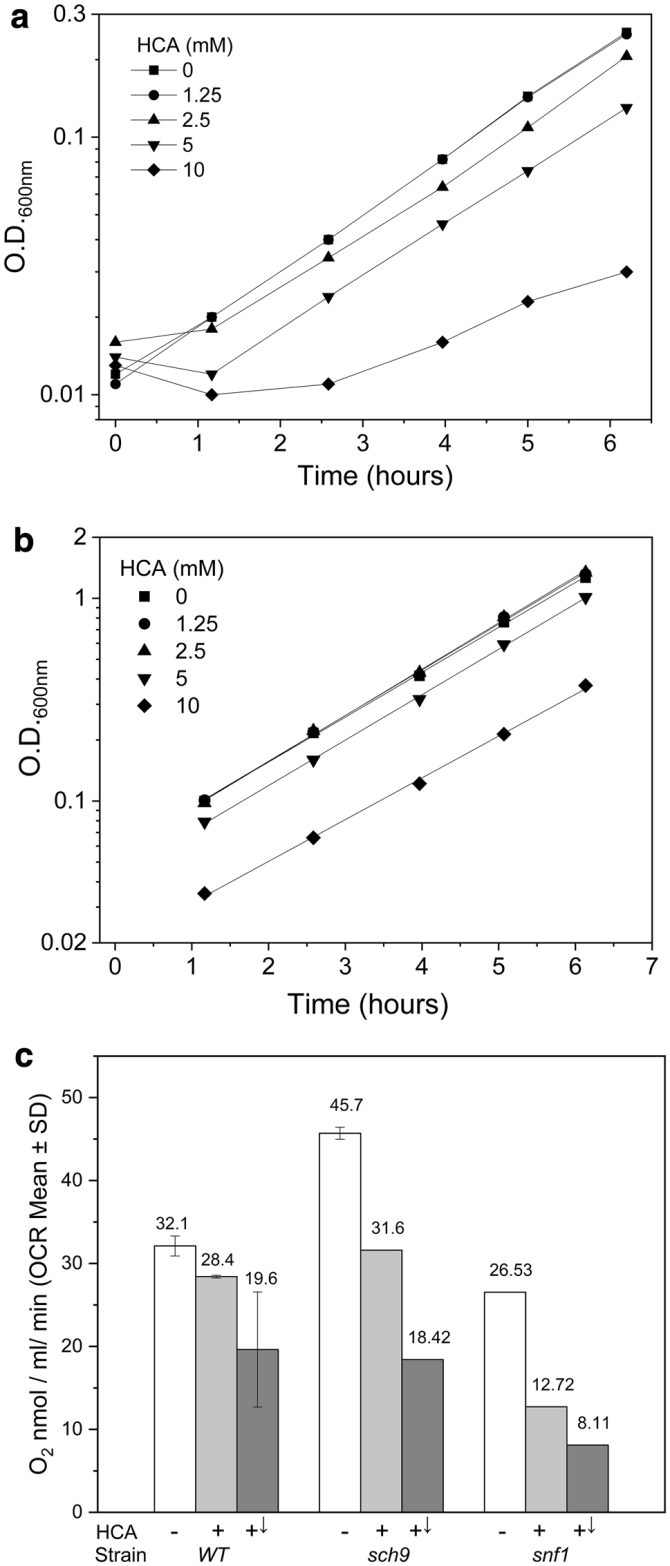
HCA affected cell growth as a function of cell physiology and inhibited yeast OCR. **a** Growth curves of HCA-treated quiescent cells after inoculation in SD glucose medium. Cells supplied with HCA resumed cell growth more slowly than control (see also Table S1). T_0_ = 17 h after cell seeding. **b** Exponential growth of yeast populations in the presence of HCA. Cells proliferated at the same exponential growth rate irrespective of the HCA presence. Doubling time of control was 81.6 min (normalized growth rates are in Table S1). **c** Oxygen consumption rate in early stationary phase cells kept in their medium during the measurements. Cells were untreated (−) or treated with 5 mM HCA from inoculum throughout their growth (+); an aliquot of these treated cells was even challenged with further 5 mM HCA just before the assay (+↓; final concentration 10 mM HCA) (n = 2)

In the previous section we have described how some genetic elements able to change yeast CLS could interact with HCA. We also observed that HCA did not alter the exponential growth rate (µ) of yeast populations but it delayed the recovery of growth of quiescent cells (Fig. 4a, b; Table S1). This appears to be consistent with the interaction between HCA and the signalling pathways of *RAS2* or *SCH9*. Indeed, in optimal growth conditions, the loss of either gene poorly affects µ [43, 44], while these regulators are important for cell responses during physiological transitions, in particular involving nutrients, cellular quiescence or oxidative stresses [9, 44–47]. Consequently, HCA effects on metabolism were more directly investigated by measuring the oxygen consumption rate (OCR) of stationary phase cells, just before they started to age (day 0 CLS, see above) (Fig. 4c). It resulted, that chronic exposure to 5 mM HCA (from cell seeding) down-regulated yeast OCR and this inhibitory response was quickly exacerbated when a further HCA dose (5 mM) was supplied to the cells. A similar marked repression was observed in both *WT* and *snf1* mutant, the latter fully responding to HCA also in CLS assays (see above). However, importantly HCA induced a strong OCR repression in *sch9* strain as well.

## Discussion

It has been proposed that bona fide CRMs should also have anti-aging properties, promoting longevity as it does a true calorie restriction regime (CR) [1]. As far as we know this is the first time that such a property was demonstrated for hydroxycitrate (HCA). It appears that HCA could largely prevent some degenerative processes leading to the fatal activation of apoptotic and necrotic pathways after an unrestricted period of growth on high glucose concentrations. An especially important example of such processes during cellular aging is represented by the excessive accumulation of ROS, associated to cell viability loss [41, 48–50]. Indeed, here we show that HCA was able to limit the accumulation of ROS in aged yeast cells challenged with hydrogen peroxide. In addition, it could also greatly limit the cell mortality even in cells still producing high ROS levels, according to a more direct effect on the apoptotic/necrotic processes. This latter possibility is strongly supported by its rescue ability from cell death induced with high concentrations of acetic acid.

Genetic analyses indicate that *ras2Δ* and *sch9Δ*, two CLS mutations extending longevity [47, 51], were partially epistatic to HCA anti-aging effects. At a first approximation this suggests that HCA can act in part by impinging on the signalling pathways driven by these elements, as also supported by the strong resistance to acetic acid or H_2_O_2_ common to *ras2*, *sch9* [37, 47, 52] and HCA-treated cells. Further, the loss of *SCH9* was almost fully epistatic to HCA treatment during the cellular response to a severe oxidative stress. However, the residual amount of *sch9* cells sensitive to oxidative stress (∼ 10%) were still rescued from cell death by the treatment with HCA. The rescue effect was even more evident in the *ras2* mutant although it was more resistant than *WT* when treated with H_2_O_2_: in our extreme conditions, HCA could save ∼ 25% of the population (for a comparison, in the same conditions ∼ 50% of WT cells were saved by HCA administration). Finally, *sch9Δ* mutation was not epistatic at all to the OCR changes induced by the HCA. Indeed, the *sch9* mutant showed a full sensitivity to the HCA-mediated partial inhibition of respiration, just like it was observed with *WT* or *snf1* cells. In conclusion, a partial effect of HCA as a molecule modulating Ras2 and Sch9 activities cannot be ruled out, or HCA might be able to modify some targets shared with these regulatory elements. However, it might primarily induce an adjustment of metabolism then sensed by Sch9 and Ras2 as well as other possible regulatory elements, so affecting fundamental nutrient and stress signalling pathways in an indirect way.

Biochemical in vitro and in vivo analyses on hydroxycitrate performed in the past support more this latter hypothesis, since HCA was found capable to either inhibit or activate several metabolic enzymes (Table S2; partially reviewed in [20, 53]). The enzymes studied belonged to vertebrate systems but they all are conserved in *S. cerevisiae*, with the exception of ACLY. In addition, many of them are allosterically controlled by citrate (CA) or citrate is part of the specific reactions they catalyse (Table S2). Indeed, citrate is a crucial sensor and regulator of cell energy level. It can control catabolic pathways through the negative feedback inhibition of key regulatory enzymes of glycolysis (e.g. phosphofructokinase 1 and 2) and TCA cycle (e.g. pyruvate dehydrogenase), so adjusting ATP production reviewed in [54, 55]). At the same time, citrate controls ATP-consuming anabolic pathways by stimulating key enzymes of gluconeogenesis (fructose-1,6-bisphosphatase) [56] and fatty acid synthesis (acetyl-CoA carboxylase) [57, 58]. Given that HCA can inhibit phosphofructokinase 1, pyruvate dehydrogenase, citrate synthase, aconitase and isocitrate dehydrogenase and might have other control activities in common with citrate (Table S2; [17–20, 53, 59, 60]), HCA itself could negatively control both glycolysis and TCA cycle.

In principle, the above picture could account for the HCA-induced inhibition of OCR and the delay of growth recovery of stationary phase cells. Of note, the exponential growth rate was unchanged by the same HCA concentrations, suggesting that the molecule was able to modulate and likely optimize the metabolic pathways rather than repressing them. We hypothesize that this metabolic control can be directly involved in the pro-longevity/anti-apoptotic properties of HCA, similarly to the effects of low glucose CR. The observation that the product of PFK1, fructose 1,6 bisphosphate, can activate the pro-aging and pro-apoptotic Ras2 protein [61] strongly support this picture. In addition, the HCA-dependent inhibition of glycolysis and TCA cycle is expected to deplete the AcCoA pools even in the absence of ACLY. In turn, AcCoA depletion can repress protein acetylations [1, 62] and activate autophagy, both in yeast and mammals [63, 64]. It is known that these effects can crucially improve life span [33], and they have been proposed as key markers of bona fide CRMs [1, 65]. In addition, HCA might also favour the yeast autophagic cascade and longevity through AcCoA carboxylase activation. Indeed, the completion of autophagy is facilitated by ACC-driven changes of cellular lipid profile and the chemical inhibition of Acc1 (but not of its activated version) accelerates chronological aging [66].

In conclusion, in yeast HCA produces dramatically positive effects on cell resistance to aging and stresses despite the lack of ACLY, possibly by directly impinging on metabolism, hence lowering the signals controlling at least Ras2 and Sch9 driven pathways. In higher eukaryotes, ACLY inhibition with HCA must also increase the citrate levels [67]. Our model system challenged with HCA together with previous biochemical studies suggests an integrated model of HCA effects (Fig. S11). It predicts that HCA can deplete AcCoA pools by repressing ACLY, as largely known, together with a HCA/citrate driven negative modulation of metabolism. In the same model, in yeast and perhaps even in higher eukaryotes, HCA might favour autophagy not only by lowering AcCoA levels but also via ACC activation, according to recent findings [68]. These working hypotheses will be investigated by directly studying HCA effects on AcCoA metabolism, protein acetylations and autophagic processes. Then the model will be preliminarily tested in *S. cerevisiae* cells expressing ACLY [29, 69] and, more conclusively, in mammalian cells.

## Materials and methods

### Yeast strains, culture conditions and media

Yeast strains are listed in Table 1.

Cells were grown at 30 °C with shaking (200 rpm) into liquid synthetic defined media (SD) containing 2% glucose, 6.7 g/l YNB w/o amino acids (ForMedium™, UK) and the proper selective drop-out CSM (Complete Synthetic Medium, ForMedium™), with the exceptions of *snf1* or *tps1* strain grown in SD + 5% glucose or 2% galactose, respectively. Growth was monitored either by measuring the absorbance ad 600 nm (OD_600_), or by counting the cell number with a Coulter Counter (Beckman-Coulter Z2) after a mild sonication [9].

### Aging experiments and cell viability

We used one of the established procedures to measure CLS, as described by Fabrizio and Longo [35], growing cells in SD (2% glucose) and prolonging incubation in their original medium. Chronological aging was monitored after arrest into stationary phase (day 0 CLS) as progressive loss of cell viability. Colony forming units (CFUs) were used to monitor viable cells. The biological replicates of the CLS experiment were 3, giving similar results. (n = 3 for each CFU). In experiments also described in following sections samples were taken in triplicate (n = 3) and experiment replicates were at least 3 if not otherwise specified.

### Flow cytometer analyses

#### Annexin V and propidium iodide (PI) staining

Protocols used were basically as those previously described [72, 73] and are more detailed in Supplementary Material and Methods. Spheroplasts of stationary phase/aging cells were obtained by incubating yeast cells in washing buffer containing 200 units/ml Zymolyase 100T (Nacalai Tesque, Inc.) for 60 min at 30 °C. Samples were analysed with a flow cytometer (CytoFLEX©, Beckman Coulter, Inc.).

#### Dihydrorhodamine (DHR) 123 and propidium iodide (PI) staining

Cells suspended in PBS were stained with DHR123 (Sigma) (final concentration 5 µg/ml) for 120 min at 30 °C in the dark, with gentle shaking. Next, PI (3 µg/ml) was added for 15 min at *r.t.* and samples were immediately analyzed with flow cytometer (CytoFLEX©).

### Acetic acid induced apoptosis

10^8^ cells per treatment of exponentially growing cells (0.6–1.0 × 10^7^ cells/ml) were collected by centrifugation, suspended in 0.5 ml of sterile water, mildly sonicated and transferred to SD (2%) at pH 3 [39] to obtain a final cell density of 10^7^ cells/ml. In cultures that had been treated with HCA (10 mM) the molecule was added again into the new medium at pH 3 at the same concentration. Cell suspension was divided in aliquots for incubation (30 °C) with or without the addition of 80 mM acetic acid. After incubation (till 200 min), CFUs were determined and/or spheroplasts were prepared for Annexin V/PI staining.

### Hydrogen peroxide induced cell death in aged cells

5 × 10^7^ Cells were collected after 1, 2 or 3 days CLS plus or minus 10 mM HCA and sonicated. After, H_2_O_2_ at the final concentration of 70 mM was added and cells were incubated for 20 min at 30 °C while gently shaken. H_2_O_2_ exposure was stopped by centrifugation and resuspension in 0.5 ml PBS buffer. Staining with DHR123/PI was performed as described above.

### Oxygen consumption rate

Oxygen consumption rate (OCR) was measured with an oxygen electrode system (Hansatech Instruments Ltd, GB). Oxygen consumption was detected polarographically by a S1 Clark-Type electrode in a 1 ml liquid-phase reaction vessel following the manufacturer instructions. Basal OCR and rates induced by HCA addition to the polarographic chamber were normalized to total protein content (n = 2; replicates = 2).

## Electronic supplementary material

Below is the link to the electronic supplementary material.

Supplementary file1 (PDF 1680 kb)

## Data Availability

The data and materials generated during the study are available from the corresponding authors.
