# Supplementary material for: In S. cerevisiae hydroxycitric acid antagonizes chronological aging and apoptosis regardless of citrate lyase
Source: Apoptosis. 2020 Jul 14;25(9):686–96. doi: 10.1007/s10495-020-01625-1 (PMC7527365; doi:10.1007/s10495-020-01625-1)
Supplement: Supplementary file 1 — Supplementary file1 (PDF 1680 kb) [file 10495_2020_1625_MOESM1_ESM.pdf]

In *S. cerevisiae* hydroxycitric acid antagonizes chronological aging and apoptosis regardless of citrate lyase

Supplementary Figures

Figure S1

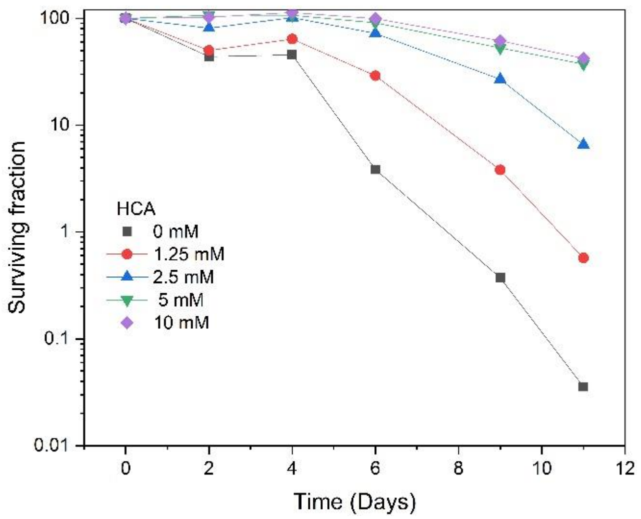

Supplementary Figure 1. Yeast chronological life span, shown as surviving cell fraction, in the presence of different concentrations of Hydroxycitric acid. The figure refers to the same data of Fig. 1a normalized on the cell number at Day 0 in each condition (put as 100%).

Figure S2

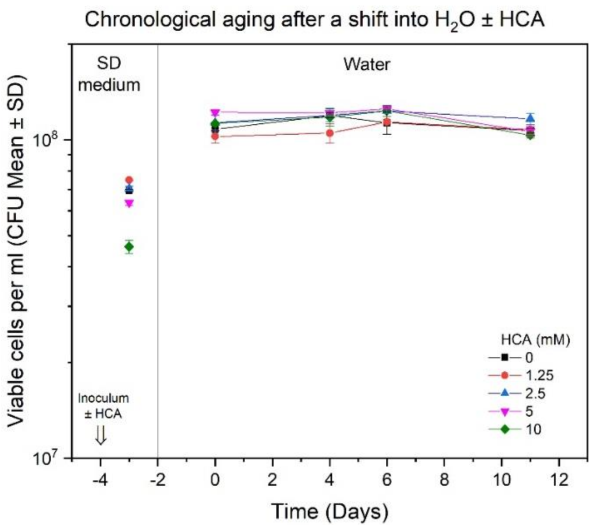

Supplementary Figure 2. At Day 0, an aliquot of the populations described in Fig.1a was shifted in water from their original synthetic glucose medium and CLS was monitored as CFUs. During 11 days in this condition, cells do not show any loss of viability, as previously described (Fabrizio P, Longo VD (2007) The chronological life span of *Saccharomyces cerevisiae*. *Methods Mol Biol* 371:89-95. doi:10.1007/978-1-59745-361-5_8)

Figure S3

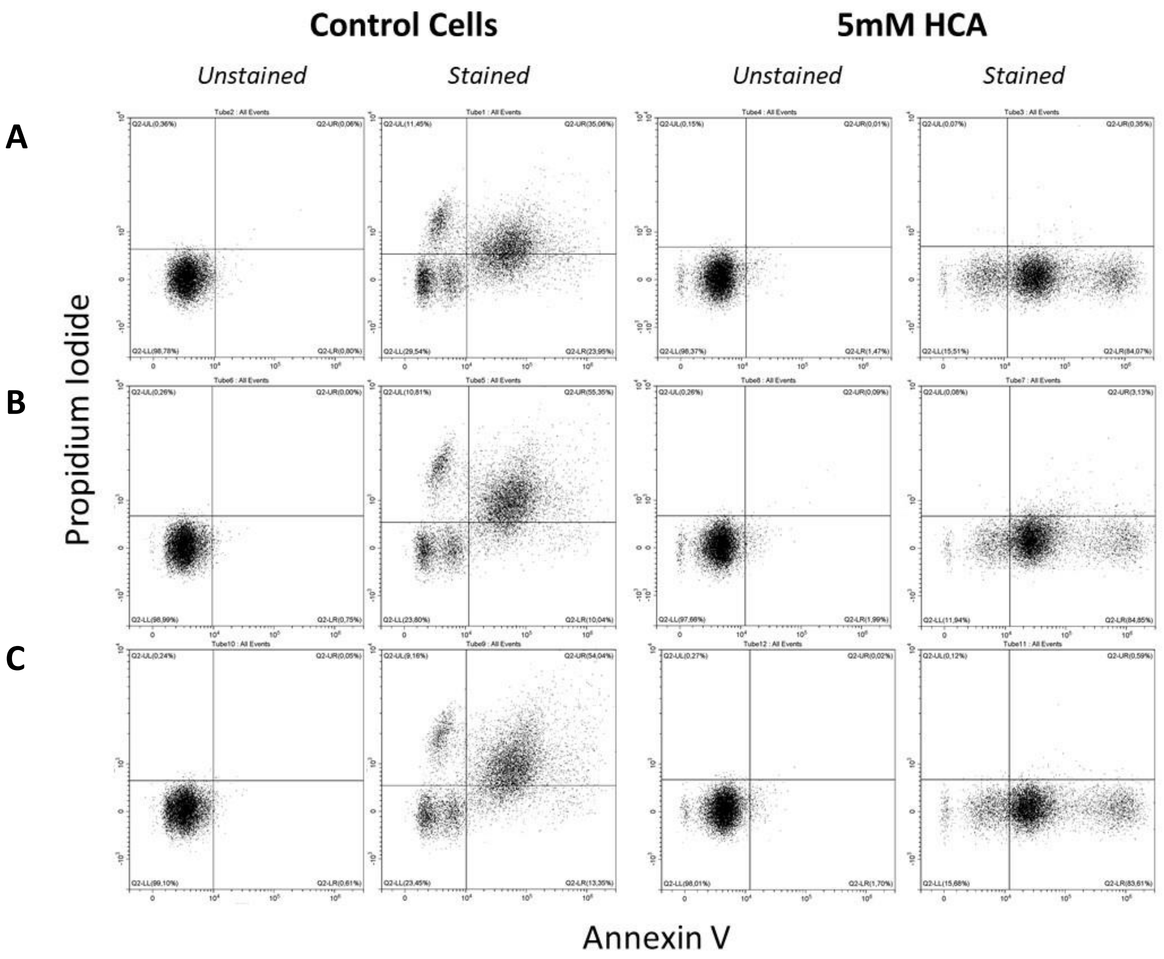

Supplementary Figure 3. Different experiments (panels A-C) showing examples of citofluorimetric analyses of apoptotic/necrotic cells performed on day 7.5 CLS (see main text for a description). Cells were treated or not with 5mM HCA, as marked in figure. Double negative live cells are located in the lower left (LL) quadrant; early apoptotic Annexin V^{+}/PI^{-} cells are in the lower right (LR) quadrant; late-apoptotic Annexin V^{+}/PI^{+} cells are in the upper right (UR) quadrant; necrotic Annexin V^{-}/ PI^{+} cells are positioned in the upper left (UL) quadrant.

Fig. S4

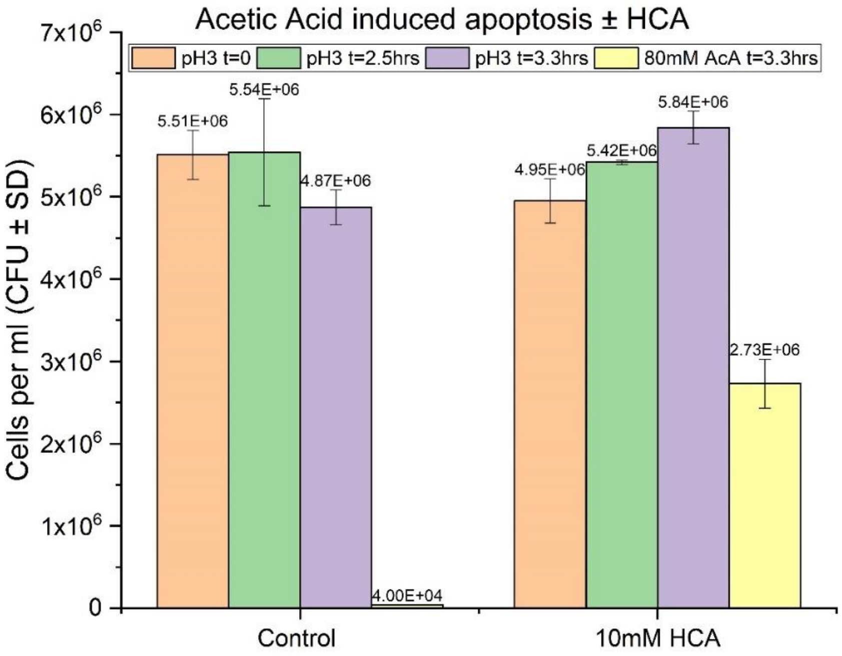

Supplementary Figure 4. Acetic acid (AcA) induced apoptotic cell death ±HCA. CFUs generating the normalized values of Fig.2a are plotted in the figure (here time is given in hours and an intermediate time is also added). See main text and Material and Methods for details.

Fig. S5

A

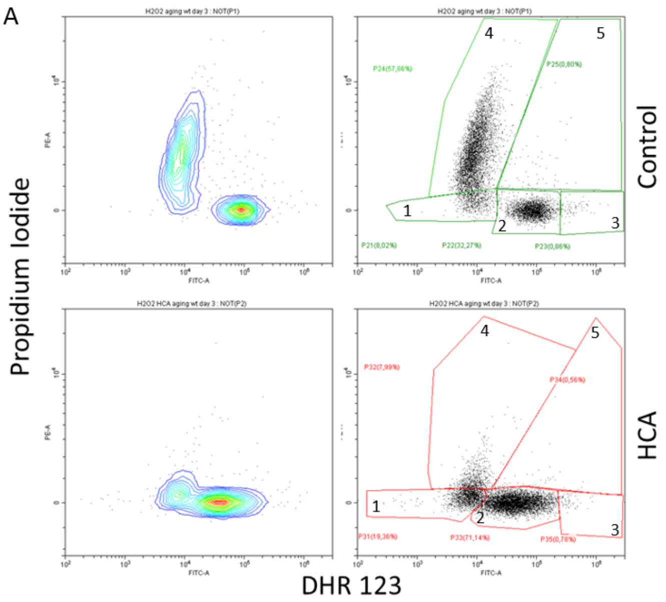

B

	Control	10mM HCA	
Classes of cells	Percent		**Cell Features**
1) $PI^-DHR123^-$	**8.02**	**19.36**	Live<br>low ROS
2) $PI^-DHR123^+$	**32.27**	**71.14**	Live<br>high ROS
3) $PI^-DHR123^{++}$	0.86	0.78	Live<br>very high ROS
4) $PI^+DHR123^-$	**57.86**	**7.99**	Dead<br>(low ROS)
5) $PI^+DHR123^+$	0.80	0.56	Dead<br>(high ROS)

Supplementary Figure 5. Representative flow cytometric analyses of DHR 123/PI double stained WT cells after exposure to H_2O_2 ±10mM HCA. **Panel A** Data are the same of Fig. 2d but are reported here as both contour or dot plots. In addition, here the distributions are showed after discharging most doublets (see supplementary M&M below) and used for the identification of different cell classes by manual gating (unstained samples used as reference are not shown). **Panel B** Cell frequencies of each class calculated after gating the distributions, automatically calculated with CytExpert Program (see M&M below).

Figure S6

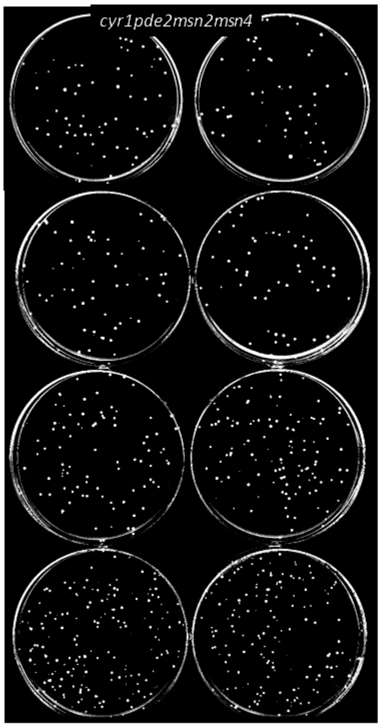

HCA (mM)	Dilution Before Plating (100µL)	Colony Number per plate (Mean±SD)	CFU/ml
0	1:10^2	64 ±7.071068	6.40x104
2.5	1:10^3	61± 2.828427	6.10x105
5	1:10^3	105± 16.97056	1.05x106
10	1:10^3	178± 12.72792	1.78x106

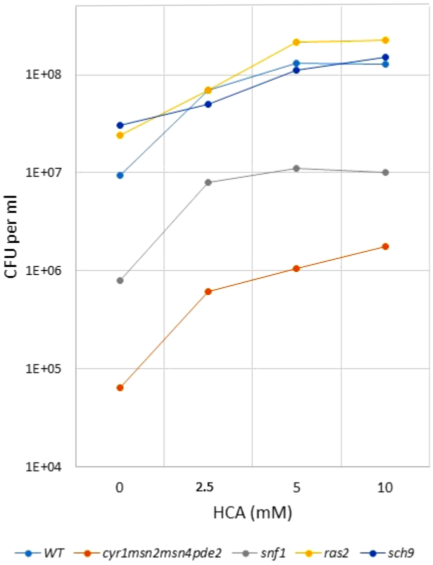

Supplementary Figure 6. WT and mutant cells were plated after 6 days of chronological aging ±HCA at different concentrations, and CFUs were counted after 3 days on YPD plates. Raw data for *cyr1msn2msn4pde2* strain are shown as an example (left part). Cells per ml in original flasks were calculated and potted for each strain (right part). These data were used to calculate the normalized cell fraction of Figs. 3a and S7

Figure S7

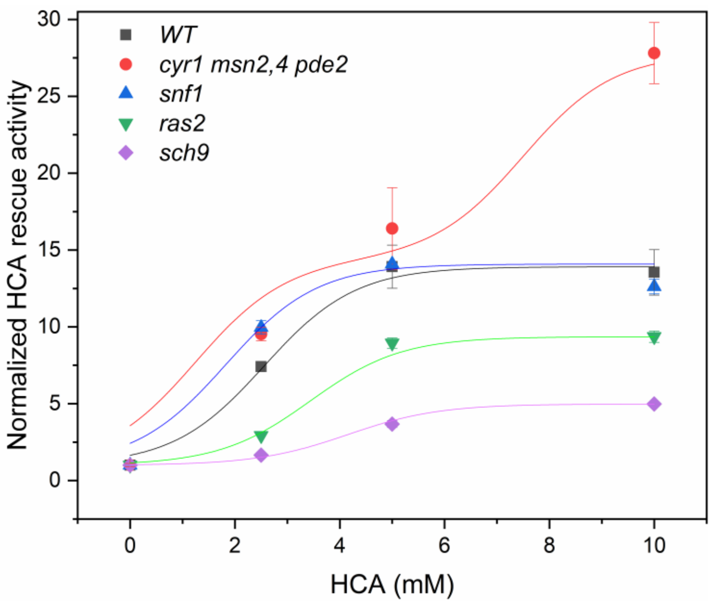

Supplementary Figure 7. Dose-response fits of the same data shown in Fig. 3a (Levenberg-Marquardt equation) produced in order to compare the mutant behaviours with quantitative parameters. Curiously, data got with *cyr1msn2msn4pde2* strain can be fitted by a biphasic curve, perhaps revealing two subpopulations, differently sensitive to HCA. EC_{50} (derived from location of curves along x axis) indicates the *effective concentration* (mM) giving a 50% increase of the rescue activity, it was: 2.55 (*WT*), 1.32 and ~7.5 (*cyr1msn2,4pde2*), 1.83 (*snf1*), 3.4 (*ras2*) and 4.15 (*sch9*). The slope (change in response per unit dose) was: 3.75 (*WT*), 3,74 and 3.74 (*cyr1msn2,4pde2*), 3.75 (*snf1*), 2.17 (*ras2*) and 1.05 (*sch9*). The maximal efficacy (greatest attainable response) was :13.92 (*WT*), ~14 and >26 (*cyr1msn2,4pde2*), 14.09 (*snf1*), 9.36 (*ras2*) and 4.98 (*sch9*). Concentration values (x axis) were plotted in a linear scale for the sake of clarity.

Figure S8

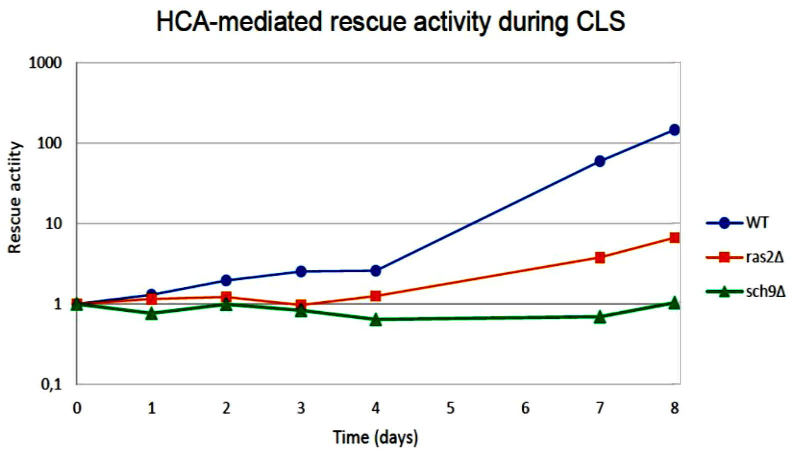

Supplementary Figure 8 Rescue activity of HCA (10mM) observed during chronological aging kinetics in minimal medium of mutant and isogenic wild type cells. Rescue activity of HCA is calculated as the ratio of survival fractions with or without HCA, at each time.

Fig. S9

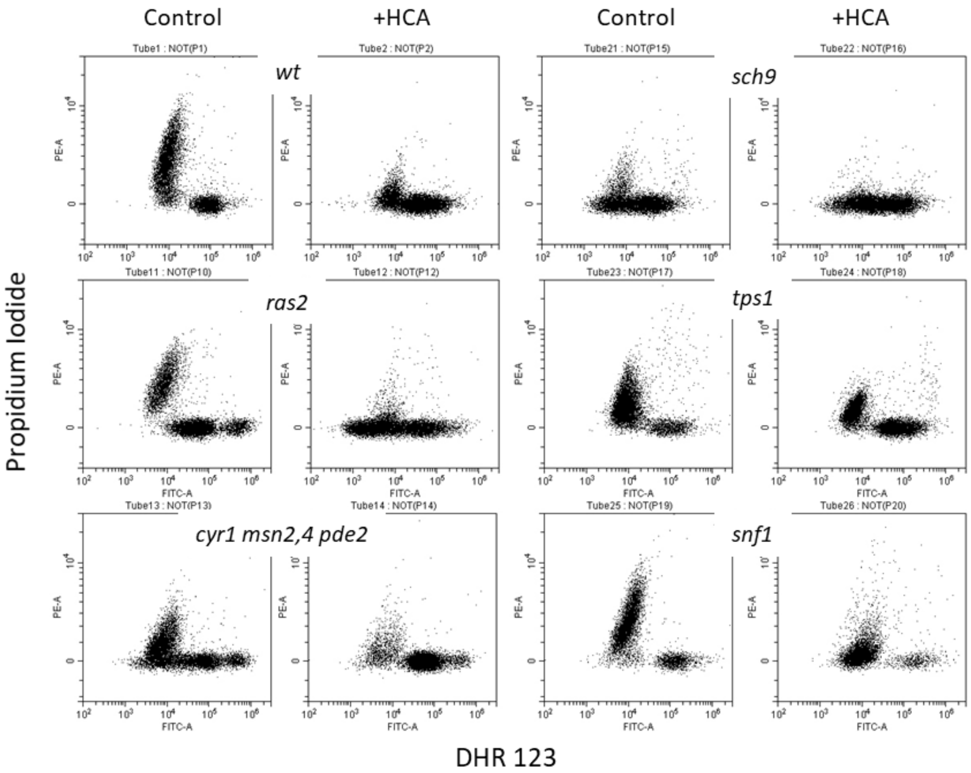

Supplementary Figure 9. Representative flow cytometric analyses of DHR123/PI stained WT and mutant cells after a severe H_2O_2 driven oxidative stress ±10mM HCA. Distributions are the same of Fig. 3b except that cell doublets were subtracted here. These corrected distributions were used for the quantitative analyses reported in Fig. 3c.

Fig. S10

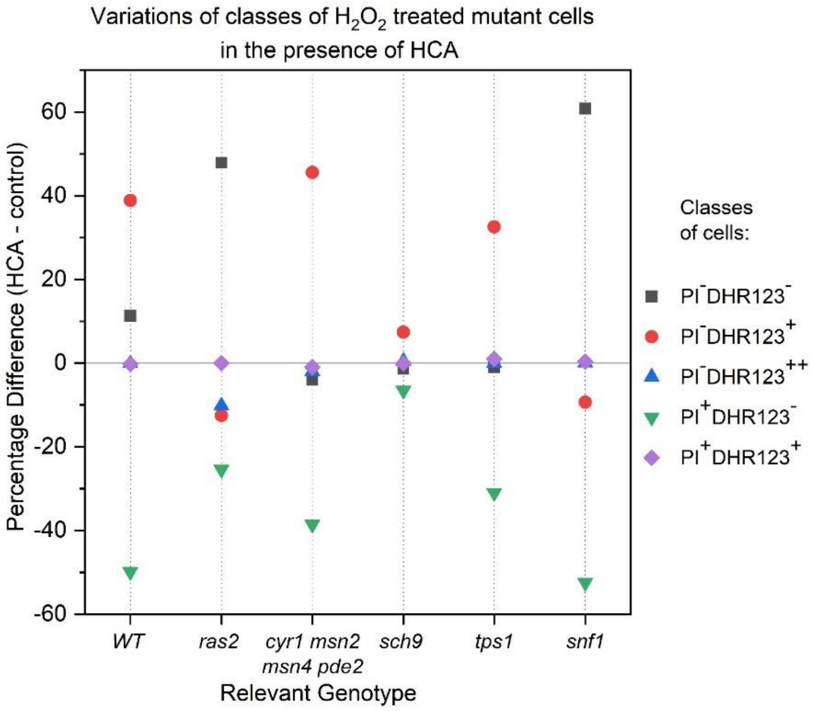

Supplementary Figure 10. Variation of cell frequency within each PI/DHR123 staining class when cells were treated with HCA, during a severe oxidative stress. The percentage of control cells in each class was subtracted to the percentage of HCA-treated cells in the same class, and the results were plotted for all mutants. See Main Text for a further description.

Figure S11

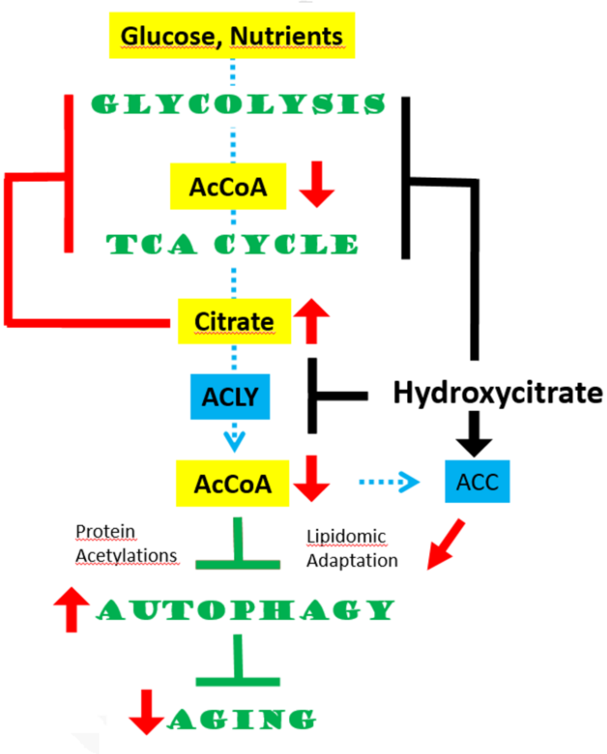

Supplementary figure 11. An integrated model of HCA biochemical effects. In this speculative model HCA can deplete AcCoA pools with ACLY-dependent and ACLY-independent mechanisms, eventually inducing autophagy. In addition, it can also favour autophagy through AcCoA carboxylase activation. Direct effects of HCA on ACLY and other metabolic enzymes are marked in black (bars and arrow) whereas the indirect effects on metabolites and cell processes are marked in red (bars and arrows). An important HCA output is the increase of longevity (demonstrated in the present study even in the absence of ACLY) that is linked to autophagy activation in the model. Blue dotted arrows indicated metabolic fluxes

Supplementary Tables

Table S1. HCA inhibited the time of growth recovery of deeply quiescent cells but not their exponential growth rate. Parameters are calculated from growth curves of Fig.4 a, b and their replicates (not shown).

	Difference in growth recovery time* (min)			Normalized specific growth rate[§] (μ)		
**HCA (mM)**	n	**mean**	SD	n	**mean**	SD
**0**	2	**0**	±0	3	**1**	±0.0197
**1.25**	2	**0**	±0	3	**1.035**	±0.018
**2.5**	2	**15.5**	±21.92	2	**1.081**	±0.01414
**5**	2	**54.5**	±34.64	2	**1.0375**	±0.03606
**10**	2	**162.5**	±45.96	3	**0.933**	±0.0251

*Differences of Lag Time respect to untreated control (0mM HCA); [§]Taken when $0.1 \leq OD_{600} \leq 1$

Table S2. Enzymes known to be inhibited or activated by citrate and/or hydroxicitrate*

ENZYME	ORGANISMS[§]	CA	HCA	PROCESS
Phosphofructokinase 1(PFK1)	v, y	↓	↓	Glycolysis
Phosphofructokinase 2(PFKFB2)		↓	**?**	
Pyruvate kinase M2(PMK2)		↓	**?**	
Pyruvate dehydrogenase(PDH)		↓	↓	TCA cycle
Citrate synthase (CS)		**p**	↓	
Aconitase (Ac)		**s**	↓	
Isocitrate dehydrogenase (IDH)		**?**	↓	
Succinate Dehydrogenase (SDH)		↓	**?**	
Cytoplasmic ATP-dependent citrate lyase (ACLY)	v	**s**	↓	Acetyl-CoA production
Nuclear ATP-dependent citrate lyase (ACLY)		**s**	**?**	Acetyl-CoA production
Fructose-1,6-bisphosphatase(FBP)	v, y	↑	**?**	Gluconeogenesis
Acetyl-CoA carboxylase (ACC)		↑	↑	Lipogenesis

*See main text for description and references. [§]v, vertebrates; y, yeast. **p**, product; **s**, substrate. Enzyme activity: ↓inhibition, ↑stimulation

Supplementary Materials and Methods

Culture conditions and media

After overnight growth cells were inoculated in parallel cell cultures (V_{medium}: V_{flask} = 1:5) at OD_{600nm} =0.005 Units without or with HCA (Sigma-Aldrich®) at the specified concentrations. Adenine concentration was always increased to 100 mg/l in culture media.

In experiments with Acetic acid (Riedel-deHaeden) the final incubation medium was adjusted at pH=3 by addition of HCl and filtered.

Colony Forming Units (CFUs) on YEPD/Agar were counted after 3 days of incubation at 30°C, in order to monitor viable cells. At day 0 the cell density based on both OD_{600} (Perkin-Elmer, Lambda EZ201, UV-VIS spectrometer) and cell number (measured using a Coulter Counter; Beckman-Coulter Z2) was used as a reference to plate, after mild sonication, about $5x10^2$ or 10^3 cells per plate, adapting serial dilutions to the progressive loss of viability. So, the absolute colony counts per plate were always ≥30 and<1000. Samples were taken triplicate for each CFU calculation. Colonies were imaged by scanning, quantified using Image (Fiji) Colony Counter plugin (https://imagej.nih.gov/ij/plugins/colony-counter.html) and their number corrected by visual inspection.

Fig.4a: Deeply quiescent cells (3 weeks in H_2O) were inoculated in SD Glucose medium. To exclude any difference in initial cell viability, live cells actually seeded were counted. CFUs per ml ±SD were 950±353.6, 1250±353.6, 950±212.1, 1000±282.8 and 950±212.1 corresponding to flasks with 0, 1.25, 2.5, 5,and 10mM HCA, respectively.

Fig.4b: Deeply quiescent cells (3 weeks on YEPD plate) were inoculated in SD Glucose medium. CFUs per ml ±SD were 7500±565.7, 7250±777.8, 8000±2262.7, 9650±777.8 and 7650±777.8 corresponding to flasks with 0, 1.25, 2.5, 5,and 10mM HCA, respectively.

Flow Cytometer analyses

Annexin V and Propidium Iodide (PI) staining. 10^8 cells per each treatment were harvested and washed twice with washing buffer (WB, Sorbitol 1 M, NaH_2PO_4 0.1 M, pH 8) at 4°C, mildly sonicated and incubated for spheroplast formation in 400µl WB containing zymolyase while gently shaking. 5 units/ml zymolyase 20T (Seikagaku Biobusiness Corporation) for 35 min at 37°C or 200 units/ml Zymolyase 100T (Nacalai Tesque Inc.) for 60 min at 30°C were used respectively for exponentially growing cells or stationary phase/aging cells. Spheroplasts were washed twice with 1 ml binding buffer (NaCl 140mM, $CaCl_2$ 2.5mM, Sorbitol 1.2M, HEPES 10mM pH 7.4), resuspended in 70µl of the same buffer and stained with 4µl PI (50µg/ml in $MgCl_2$ 5mM, RNase 5mM, Sorbitol 1.2M Tris 10mM pH 7) and 5µl Annexin V (ImmunoTools Cat No: 31490013; ready to use). After 15 min incubation at *r.t.* in the dark, samples were washed and resuspended in binding buffer before Flow Cytometer (CytoFLEX©, Beckman Coulter, Inc.) analyses. Data were collected (≥10^4 events) and plotted with CytExpert program (Beckman Coulter, Inc.).

Most doublets were discharged before the quantitative analyses of flow cytometry cell distributions, by using FCS-A (Forward Scatter-Area) vs FSC-W (FSC-Width) plots or equivalent methods. So, only the corrected distributions were used for the quantitative analyses reported in Figures. However, consistent results were obtained even without any doublet correction.

Data processing

Results were elaborated and analysed with OriginPro program (OriginLab Corporation; licensed to University of Padua).

This supplementary material is linked to the following *Apoptosis* article:

In *S. cerevisiae* hydroxycitric acid antagonizes chronological aging and apoptosis regardless of citrate lyase

By M. David Baroni[1,2,3], Sonia Colombo[2], Olivier Libens[2], Rani Pallavi[3], Marco Giorgio[3,4] and Enzo Martegani[2]

[1]University of Padua, Department of Biology, Padua, Italy

[2]University of Milano-Bicocca, Department of Biotechnology and Biosciences, Milan, Italy

[3]European Institute of Oncology (IEO), Department of Experimental Oncology, Milan, Italy

[4]University of Padua, Dept. of Biomedical Sciences, Padua, Italy

Correspondence to: M. David Baroni, mauriziodavide.baroni.unipd.it and Enzo Martegani, enzo.martegani@unimib.it
